# Combat Exposure Severity Is Associated With Reduced Cortical Thickness in Combat Veterans: A Preliminary Report

**DOI:** 10.1177/2470547017724714

**Published:** 2017-08-22

**Authors:** Lynnette A. Averill, Chadi G. Abdallah, Robert H. Pietrzak, Christopher L. Averill, Steven M. Southwick, John H. Krystal, Ilan Harpaz-Rotem

**Affiliations:** 1 Clinical Neurosciences Division, U.S. Department of Veterans Affairs National Center for PTSD, VA Connecticut Healthcare System, West Haven, CT, USA; 2 Department of Psychiatry, Yale University School of Medicine, New Haven, CT, USA

**Keywords:** combat, veteran, cortical thickness, early life stress, childhood trauma, posttraumatic stress disorder, structural neuroimaging

## Abstract

**Background:**

Chronic stress and related physiological responses are known to have deleterious effects on neural integrity. Combat exposure is a notoriously pathogenic stressor, and with over 2 million U.S. troops deployed to active combat zones since 2001, there is an urgent need to advance our understanding of its potential neural impact. Previous evidence suggests structural alterations in posttraumatic stress disorder (PTSD) and more recent studies have explored cortical thinning specifically. This preliminary study investigates the impact of combat exposure on cortical thickness, controlling for history of early life stress and age.

**Methods:**

Twenty-one combat-exposed Veterans with PTSD and 20 non-PTSD combat-exposed controls (mean age 32.7) completed the Combat Exposure Scale, Childhood Trauma Questionnaire, and structural magnetic resonance imaging in a Siemens 3T TIM trio system. General linear model was used to examine the effect of combat exposure on cortical thickness, controlling for early life trauma exposure and age using cluster-wise correction (*p* < 0.05).

**Results:**

This preliminary study found a negative correlation between combat exposure severity (CES) and cortical thickness in the left superior temporal and left rostral middle frontal regions, as well as an interaction between PTSD diagnosis status and CES, in the superior temporal/insular region showing a stronger negative correlation between CES and cortical thickness in the non-PTSD group.

**Conclusions:**

Though caution should be taken with interpretation given the preliminary nature of the findings, the results indicate combat exposure may affect cortical structure beyond possible alterations due to early life stress exposure or PTSD psychopathology. Though replication in larger samples is required, these results provide useful information regarding possible neural biomarkers and treatment targets for combat-related psychopathology as well as highlighting the pathogenic effects of combat.

## Introduction

Combat exposure is a notoriously pathogenic stressor that often precipitates the onset or worsening of posttraumatic stress disorder (PTSD), a stress-related psychiatric disorder characterized by reexperiencing, emotional and behavioral avoidance, negative cognitions and mood, and hyperarousal symptoms.^1^ The prevalence of PTSD among combat-exposed Veterans worldwide is as high as 25%, depending on service era.^2,3^ Over 2 million U.S. troops, and millions more from across the globe, have been deployed to active combat zones since 2001 creating unprecedented need to advance our understanding of the potential neurobiological consequences associated with war and combat exposure and to investigate potential treatment targets to inform novel drug development.

Preclinical and clinical research has demonstrated the negative effects of chronic stress and related physiological responses on neural tissue integrity, structural remodeling, and volumetric changes.^4–9^ Converging evidence shows that trauma and stress-induced impairment in glutamate release and glial reuptake precipitate excitotoxic levels of extrasynaptic glutamate, which contribute to the development and maintenance of the pathophysiology of stress-related disorders, such as PTSD, major depression, and generalized anxiety.^9–11^ These stress-induced neuronal atrophy changes include reduction in dendritic arborization and spine density, as well as synaptic strength.^4,6,9,10^ These microstructural alterations are believed to underly the gray matter deficits reported in PTSD and other stress-related neuropsychiatric disorders.^7,8,10^

Several, though not all, previous structural neuroimaging studies have demonstrated gray matter abnormalities in PTSD, in particular reduced hippocampal volume^7,8,12,13^ and prefrontal cortical thickness,^14,15^ in addition to mixed evidence of increased or reduced amygdala volume,^16,17^ which may be mitigated by early life stress (ELS), severity of exposure, and the time course of the disorder. Two recent studies examined the interplay between combat exposure and ELS on brain volume and thickness in Veterans^18,19^ and found that ELS may increase sensitivity to combat trauma and susceptibility to developing trauma- or other stress-related psychopathology in adulthood. The extent to which combat exposure severity affects cortical thickness, after controlling for ELS and PTSD, is not fully known. Advancing our understanding of the possible unique neural effects of combat exposure has potential to help identify biomarkers and treatment targets for early intervention and treatment.

This preliminary study examines the effects of combat exposure on cortical thickness, controlling for ELS (specifically childhood abuse/neglect) history. Based on current evidence regarding the effects of chronic stress, we hypothesized that combat exposure severity will be associated with reduced cortical thickness. We also predicted that those with a diagnosis of PTSD will demonstrate greater reductions in cortical thickness than combat-exposed healthy controls.

## Methods

### Participants

This preliminary study included 20 male combat-exposed Veterans with PTSD and 21 age-matched male combat-exposed healthy controls (combat controls; CC). All participants had been deployed on one or more tours to Iraq and/or Afghanistan and reported exposure to combat-related experiences. All participants were 18 to 50 years of age and were excluded based on moderate to severe traumatic brain injury (TBI), neurological disorder, and magnetic resonance imaging (MRI) contraindications. For those participants with PTSD, current drug and/or alcohol abuse and recent change in antidepressant medications were also exclusionary. To help increase generalizability of any findings, Veterans with a history of mild TBI and those who were on a stable dose (four weeks or more) of an antidepressant or other select medications were eligible to participate. The Yale University Human Research Protection Program and the VA Connecticut Healthcare System Human Subjects Subcommittee approved the study. All participants provided written informed consent before any procedures took place.

### Clinical Assessment

The presence and severity of PTSD was assessed using the gold standard, semi-structured Clinician-Administered PTSD Scale (CAPS-IV).^20^ In the current study, a cutoff of 50 was used to differentiate PTSD from non-PTSD. Exposure to wartime and combat stressors was assessed using the Combat Exposure Scale (CES),^21^ which classifies self-reported experiences into one of five severity groups ranging from “light” to “heavy.” ELS, specifically childhood abuse/neglect, was assessed using the Childhood Trauma Questionnaire (CTQ),^22^ a standardized, retrospective self-report comprised of five subscales—physical, emotional, and sexual abuse, and physical and emotional neglect. The Beck Depression Inventory—Second Edition (BDI-II)^23^ is a 21-item self-report measure of depressive symptoms.

### MRI Data Acquisition and Processing

MRI data acquisition was performed using a 3T Siemens TIM Trio system with a 12-channel head coil (MPRAGE, voxel size 1 × 1 × 1 mm, repetition time 2.5 s, echo time 2.77 ms, flip angle 7°). All processing procedures were completed on the same machine to achieve optimal measurement reliability across data processing conditions and to avoid discrepant findings based on computer or software specifications.^24^ MRI images carefully reviewed for motion, artifacts, and other potential problems before beginning processing and analysis. Scans were processed to create individualized models of each participant’s cortical mantle through the fully automated recon-all cortical reconstruction pipeline in the FreeSurfer image analysis suite, a widely used and freely available software program (http://surfer.nmr.mgh.harvard.edu).

Details regarding these procedures and the benefits of using an inflated surface are well described in earlier publications,^25–28^ and the reliability of obtaining quality measurements of cortical thickness from MRI scans has been well documented.^29–31^ Briefly, images processing begins with motion artifact correction and removal of non-brain tissue (i.e., skull stripping). Next the images are transformed using intensity normalization and Talairach transformation.^32^ We then complete signal-intensity correction, automated topology correction, and tessellation of the gray matter/white matter boundary,^33,34^ followed by automated segmentation of the white matter surface and reconstruction of the pial surface at the location with greatest shift in intensity defines the transition to other tissues.^26,27^ Spherical registration and surface inflation procedures are based on the matching of cortical folding patterns across individuals. Cortical thickness was computed as the closest distance from the gray matter/white matter boundary to the gray matter/CSF boundary at each vertex on the tessellated surface.^27^ In this case, using FreeSurfer’s automated pipeline cortical thickness was mapped to the inflated surface and filtered using a circularly symmetric Gaussian surface-based smoothing kernel with a full width at half-maximum (FWMH) of 20 mm and averaged across participants (see earlier works^27,35^ for more detailed procedures).

### Data Analysis

Independent *t* tests were used to examine the difference in the demographic and clinical measures between the study groups. Statistical comparisons of global data and surface maps were generated in FreeSurfer’s Query, Design, Estimate, Contrast (QDEC) application using a general linear model (GLM) whole-brain vertex-wise analysis. Given the known significant effects of aging on cortical structure and integrity,^35^ participant age was retained as covariate in all cortical thickness analyses. We conducted a GLM with Group (PTSD vs. CC) as discrete variable, CES as continuous variable, Group × CES as an interaction term, and ELS and age as covariates. This model examined the effects of CES severity, PTSD status, and the interaction between groups and CES on cortical thickness across the whole cerebrum, while controlling for ELS and age. Statistical significance levels were cluster-corrected using a Monte Carlo simulation to obtain a corrected *p* < 0.05.

## Results

### Demographics and Clinical Characteristics

Demographic variables and measures assessing PTSD diagnostic status and symptom severity, combat exposure, and ELS are provided in Table 1. Higher CES, BDI-II, and CAPS scores were found in the PTSD group. There were no significant differences between rates of emotional, physical, or sexual abuse, nor emotional or physical neglect between Veterans with PTSD and combat controls.

**Table 1.** Brief demographic, psychological, and cortical thickness information.

	Full sample (*n* = 41) Mean (SD)	PTSD (*n* = 20) Mean (SD)	CC (*n* = 21) Mean (SD)	*p*
Age	32.66 (7.86)	32.05 (7.25)	33.24 (8.55)	0.64
CES	14.76 (10.23)	19.65 (10.48)	10.10 (7.64)	0.002
CTQ-EA	9.12 (4.79)	9.90 (4.72)	8.38 (4.85)	0.32
CTQ-PA	7.83 (3.45)	8.15 (3.13)	7.52 (3.72)	0.57
CTQ-SA	5.98 (2.54)	5.75 (1.62)	6.19 (3.20)	0.59
CTQ-EN	11.59 (5.37)	11.75 (5.90)	11.43 (4.95)	0.85
CTQ-PN	7.93 (3.50)	8.30 (3.78)	7.57 (3.26)	0.51
BDI-II	13.71 (12.75)	19.80 (13.24)	7.90 (9.29)	0.002
CAPS-IV	28.51 (29.57)	52.60 (23.79)	5.57 (8.84)	0.00
CES/CT Main Effect L-SP	3.02 mm (0.16)	2.98 mm (0.15)	3.05 mm (0.16)	–
CES/CT Main Effect L-RMF	2.59 mm (0.19)	2.53 mm (0.18)	2.64 mm (0.18)	–
Interaction Group × CES L-SP/INS	3.09 mm (0.17)	3.07 mm (0.15)	3.11 mm (0.19)	–

PTSD: posttraumatic stress disorder; CC: combat-exposed healthy control; CES: Combat Exposure Scale; CTQ-EA: Childhood Trauma Questionnaire Emotional Abuse Subscale; CTQ-PA: physical abuse; CTQ-SA: sexual abuse; CTQ-EN: emotional neglect; CTQ-PN: physical neglect; BDI-II: Beck Depression Inventory; CAPS-IV: Clinician-Administered PTSD Scale; CES/CT Main Effect L-SP: average cortical thickness in the main effect of CES in the left superior temporal region; CES/CT Main Effect L-RMF: average cortical thickness in the main effect of CES in the left rostral middle frontal region; Interaction Group × CES L-SP/INS: average cortical thickness for the interaction between group and CES found in the left superior temporal and insular regions; mm: millimeters.

### Main Effects of Group and CES

**Figure 1.**
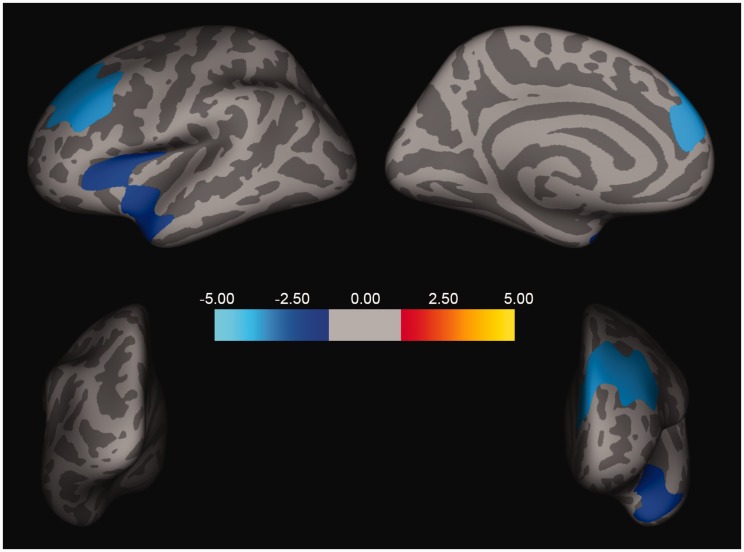
Cortical thickness and combat exposure severity. Two clusters, one in the left superior temporal region (size = 2686.35 mm^2^, xyz = −38, 16, 10; *z* = −2.12, *corrected p* < 0.05) and one in the left rostral middle frontal region (size = 3699.62 mm^2^, xyz = −37, 24, 25; *z* = − 3.70, *corrected p* < 0.05), remained significant following cluster correction. Both show a negative correlation between CES severity and cortical thickness.

**Figure 2.**
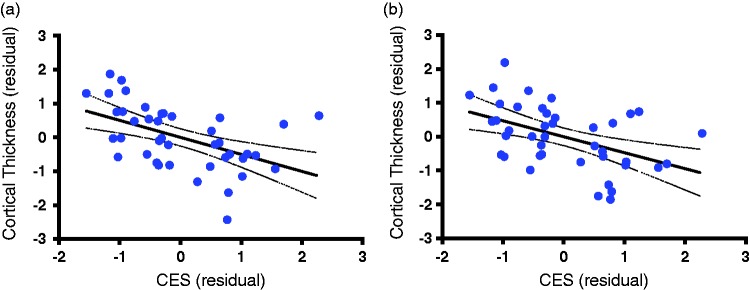
Scatter plots of cortical thickness and combat exposure severity. The scatter plots indicate the extracted average cortical thickness, showing the negative slop correlating CES and cortical thickness residuals (i.e., after regressing the effects of age, ELS, and Group (PTSD diagnosis status). (a) The scatter in the significant cluster found in the superior frontal region *(r* = −0.50, *n* = 41, *p* = 0.0008). (b) The scatter in the rostal middle frontal region (*r* = −0.47, *n* = 41, *p* = 0.0018).

Following correction for multiple comparisons using Monte Carlo Simulation, the GLM model revealed a significant main effect of CES scores but no Group effect. As shown in Figure 1, CES scores were significantly associated with two clusters showing a negative correlation between CES severity and cortical thickness in the left superior temporal (size = 2686.35 mm^2^, xyz = −38, 16, 10; *z* = −2.12, *corrected p* < 0.05) and left rostral middle frontal (size = 3699.62 mm^2^, xyz = −37, 24, 25; *z* = −3.70, *corrected p* < 0.05) regions. To illustrate the relationship between CES severity and cortical thickness at the individual level, we extracted the average cortical thickness in each of these two clusters from each subject and plotted a scatter showing the negative slope correlating CES and cortical thickness residuals (i.e., after regressing the effects of age, ELS, and Group; Figure 2(a) and (b)).

### Interaction Between Group and CES

**Figure 3.**
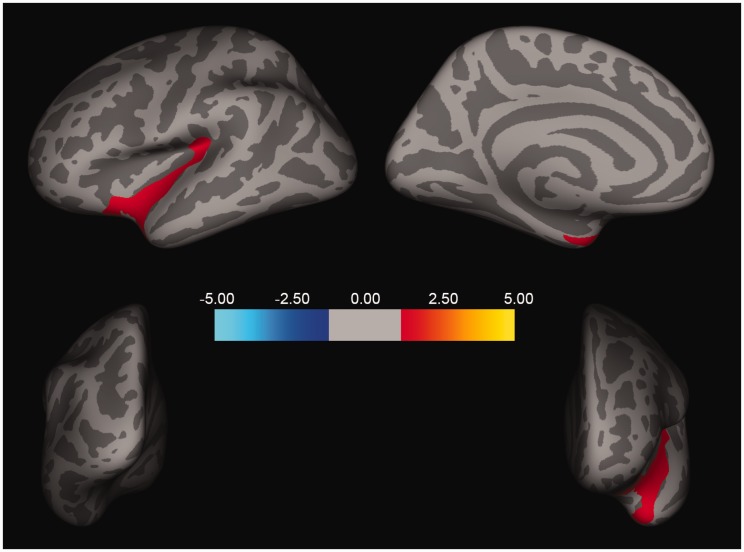
Interaction of PTSD diagnosis status/group, CES, and cortical thickness. One cluster in the superior temporal/insular region (size = 2077.41 mm^2^, xyz = −32, −32, 17; *z* = −1.47, *p* < 0.05) remained significant following cluster correction, demonstrating the interaction between group (PTSD diagnosis status) and CES. A stronger negative correlation is found between CES and cortical thickness in combat-exposed control group, compared to the PTSD group.

**Figure 4.**
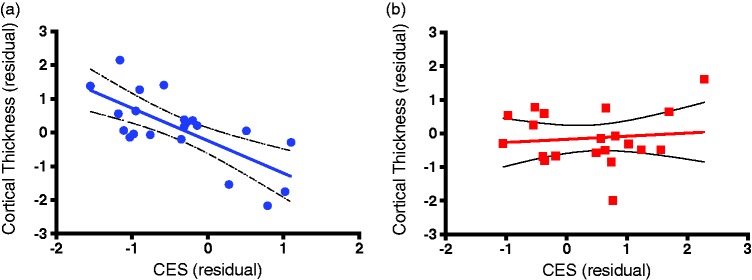
Scatter plots of CES and PTSD relative to cortical thickness. The scatter plots indicate the extracted average cortical thickness in the significant cluster from each participant. These show the correlated thickness with CES and delineate the interaction between CES severity and PTSD status. This correlational analysis showed a significant negative association between CES and cortical thickness in the CC (*r* = 0.51, *n* = 20, *p* = 0.0003; Figure 4(a)) but not in the PTSD group (*r* = 0.012, *n* = 21, *p* = 0.65). (a) Combat Control (CC) Group. (b) Veteran + PTSD Group.

Following Monte Carlo Simulation correction for multiple comparisons, the GLM model also revealed significant interaction between Group and CES in one cluster in the superior temporal/insular region (size = 2077.41 mm^2^, xyz = −32, −32, 17; *z* = −1.47, *p* < 0.05; Figure 3), showing higher negative correlation between CES and cortical thickness in the CC, compared to the PTSD group. To delineate the interaction between CES severity and PTSD status, we extracted the average of cortical thickness in the significant cluster from each participant and correlated thickness with CES in each group. This correlational analysis depicted a significant negative association between CES and cortical thickness in the CC (*r* = 0.51, *n* = 21, *p* = 0.0003; Figure 4(a)), but not in the PTSD group (*r* = 0.012, *n* = 20, *p* = 0.65; Figure 4(b)).

## Discussion

The results, demonstrating that combat exposure severity is negatively associated with cortical thickness in the left frontal and temporal cortex of combat-exposed Iraq/Afghanistan Veterans, are largely consistent with previous investigations of gray matter integrity in PTSD as prefrontal regions have been well established to play a cardinal role in the pathophysiology of both PTSD and resilience/recovery.^14,36,37^ This work is also largely consistent with previous investigations of cortical thickness in Veteran samples, also highlighting the prefrontal and superior temporal regions.^14,15,38,39^ These associations remained significant after adjustment for PTSD status, ELS, and age.

The significant negative association found between combat exposure and cortical thickness in the insular area of combat-exposed Veterans not meeting diagnostic criteria for PTSD is interesting. Though very preliminary, this result may suggest insidious neurobiological effects of combat and war-time stressors, regardless of whether or not these occur in the context of a full diagnosis of PTSD. This result is in line with previous findings suggesting the experiencing of having PTSD symptoms, even if subthreshold, may confound the neural effects of combat exposure.^15^ It is also possible that alterations in cortical GM integrity in certain brain regions may influence the expression of trauma-related symptoms.^15^

It has been well established that chronic, unpredictable stress has adverse neurobiological effects.^40^ The nature of sustained combat is a unique experience of uncontrolled and unpredictable stress, often requiring heightened awareness of self and surroundings; regular, quick manipulation and retrieval of data; and continual, often high intensity sensory input and threat assessment for prolonged periods. This is especially likely in an urban warfare environment like the combat theaters in Iraq and Afghanistan. It is possible during periods of prolonged extreme stress such as a combat deployment that these areas experience deleterious effects on neural integrity, as if they were “over-worked” or “prematurely aged” as a consequence of the need to be nearly always alert, aware, and watchful. The negative correlation is stronger in the non-PTSD group, and the data in Figure 4 portray a plateau-like effect for the PTSD group, suggesting there are perhaps vulnerability factors or other differences inherent to those who develop PTSD compared to those who do not. This underscores the need for further analysis of the role of the insula in trauma processing and the development of PTSD. Further, as previous work has implicated prefrontal regions in both the pathophysiology of PTSD and resilience, it will be important to further explore the potential role of the superior temporal and insular regions as aspects of vulnerability/resilience.

Finally, in this relatively small cohort, PTSD status was unrelated to cortical thickness after Monte Carlo correction for multiple comparisons and controlling for CES, ELS, and age (GLM Group main effect) suggesting that prior significant stress may potentially account for some the observed cortical thickness in veterans reported in other studies.

The brain regions surviving correction for multiple comparisons, identified in relation to CES severity and reduced cortical thickness are associated with functions that may contribute to symptoms of stress-related psychopathology. The rostral middle frontal region is thought to be involved in self- and other-awareness in coordination with sensory and emotional processing, working memory, fear conditioning, selective responding, and stimulus-orientated thought.^41^ The superior temporal region is associated with speech perception, pattern classification, and auditory processing and specialized encoding.^42^ If we consider the way fear-evoking memories seem to be encoded and recalled in the context of stress-related psychopathology, and the potential auditory trauma-triggers in reexperiencing and avoidance (e.g., the sound of a helicopter or gun fire) alterations or damage in this area could potentially serve as a significant factor for some of the well-documented characteristics of combat-related PTSD. The insular cortex is associated with self-awareness specifically of one’s visceral and internal states including autonomic physiological responses such as heart rate and respiration.^43,44^ It is possible that alternations in this area may compromise the capacity for regulation of physiological hyperarousal that is a common response to fear.

This investigation is preliminary in nature and has limitations that must be acknowledged. First, the sample size is relatively small and comprised solely of male combat veterans. Larger and more diverse samples will be necessary to ascertain generalizability of the findings and to optimize our ability to detect a main effect of PTSD. Especially as women are increasingly involved in combat roles, being able to explore sex differences will be important in prevention and treatment planning. Combat severity in this sample is somewhat limited and a broader range of exposure severity will provide rich data regarding the neural consequences of this type of chronic stress. Mild TBI (mTBI) was not an exclusionary criteria and may have contributed to cortical alterations.

Areas for future research include impact of multiple deployments; type and duration of combat exposure and combat severity; PTSD symptom cluster analysis; impact of blast exposures, even in the absence of TBI; time since deployment (to advance our understanding of the time course of cortical alterations); longitudinal data that would allow for assessment of pre- and post-deployment to further characterize the effects of combat; examination of cognitive capacity and function as it is possible there is reverse causality in which lower cortical thickness is associated with increased likelihood of serving in combat roles; assessment of nonmilitary adult trauma; assessment of types of ELS and trauma not assessed by the CTQ; careful examination of potential confounds including other psychopathology, substance abuse, medications, and physical/medical conditions; other possible vulnerabilities and protective factors; factors that may be relevant to military personnel more likely to see sustained combat or multiple deployments (e.g., education level, personality characteristics). Relative to the comment about physical/medical conditions, a recent study found that chronic pain may influence the relationship between combat exposure and cortical thickness in Veterans.^40^

Despite these limitations, results of this study indicate that combat exposure is associated with reduced cortical structure beyond possible alterations due to ELS exposure or PTSD, highlighting the need for careful assessment of combat exposure in returning Veterans. Though very preliminary and thus requiring caution in interpretation, the results further highlight the pathogenic effects of combat, regardless of whether or not these experiences lead to the development of PTSD. These brain regions are associated with memory, response suppression, and emotion dysregulation. The nature of combat, requiring heightened awareness of self and surroundings, continual sensory input, and regular manipulation/retrieval of data, may influence the demonstrated structural alterations. Reductions in cortical thickness may be secondary to alterations in the gray/white matter boundary related to a loss of dendrites, reduced dendritic spines, or differences in myelination within specific brain regions,^38,40^ or reduced or altered glial cell production, density, and function.^10^ Future, multimodal assessment may provide insights into neural biomarkers and treatment targets that can advance our understanding of the neurobiology of chronic exposure to traumatic stress and inform prevention, intervention, and treatment including novel drug development.
